# Structural Insights into the Role of β3 nAChR Subunit in the Activation of Nicotinic Receptors

**DOI:** 10.3390/molecules27144642

**Published:** 2022-07-20

**Authors:** Petros Giastas, Athanasios Papakyriakou, George Tsafaras, Socrates J. Tzartos, Marios Zouridakis

**Affiliations:** 1 Department of Neurobiology, Hellenic Pasteur Institute, GR11521 Athens, Greece; tsafarasgeorgios@gmail.com (G.T.); stzartos@gmail.com (S.J.T.); 2 Department of Biotechnology, Agricultural University of Athens, GR11855 Athens, Greece; 3 Institute of Biosciences and Applications, NCSR “Demokritos”, GR15310 Athens, Greece; thpap@bio.demokritos.gr

**Keywords:** nAChR, pentameric ligand gated ion channels, pentamers, principal binding site, complementary binding site, α4β2 nAChR, α4β2β3 nAChR, electrophysiology, molecular dynamics, crystal structure

## Abstract

The β3 subunit of nicotinic acetylcholine receptors (nAChRs) participates in heteropentameric assemblies with some α and other β neuronal subunits forming a plethora of various subtypes, differing in their electrophysiological and pharmacological properties. While β3 has for several years been considered an accessory subunit without direct participation in the formation of functional binding sites, recent electrophysiology data have disputed this notion and indicated the presence of a functional (+) side on the extracellular domain (ECD) of β3. In this study, we present the 2.4 Å resolution crystal structure of the monomeric β3 ECD, which revealed rather distinctive loop C features as compared to those of α nAChR subunits, leading to intramolecular stereochemical hindrance of the binding site cavity. Vigorous molecular dynamics simulations in the context of full length pentameric β3-containing nAChRs, while not excluding the possibility of a β3 (+) binding site, demonstrate that this site cannot efficiently accommodate the agonist nicotine. From the structural perspective, our results endorse the accessory rather than functional role of the β3 nAChR subunit, in accordance with earlier functional studies on β3-containing nAChRs.

## 1. Introduction

Nicotinic acetylcholine receptors (nAChRs) belong to the superfamily of pentameric ligand gated ion channels that are widely expressed in the neuromuscular junction (muscle type), in the central and peripheral neurons as well as in other tissues (neuronal types), and are naturally activated by the neurotransmitter acetylcholine (ACh) [1,2,3]. Their overall symmetry is mostly pseudo-pentameric as they are assembled in various stoichiometries by the combination of five subunits selected from a repertoire of 16 homologous subunits [4]. Thus, they form heteropentamers, with the exception of α7 and possibly α9 nAChRs which can form homopentamers as well, by the assembly of five identical subunits [5]. Each nAChR subunit is organized in three domains, (a) the extracellular (ECD) (205–235 aminoacid residues long), (b) the transmembrane, comprising four membrane-spanning α-helices and (c) the intracellular, which varies significantly in size among the subunits and is considered mostly unstructured [6]. The ACh binding site, usually referred to as an orthosteric binding site, is located at the interface between the ECDs of two adjacent subunits, one contributing the principal or (+) side (consisting of loops A, B and C) and the other one contributing the complementary or (−) side (consisting of loops D, E and F) [6,7]. While in homopentameric nAChRs the binding site lies inevitably between two α subunits, in heteropentameric neuronal nAChRs, the binding site is formed by one α subunit, contributing the principal side, and either a β or an α subunit, contributing the complementary side. In particular, electrophysiological studies of various neuronal nAChR stoichiometries heterologously expressed in *Xenopus* oocytes or in mammalian cell lines have shown that the (+) side of the binding site consists of one of the α2, α3, α4, α6 or α7 subunits and that the (−) side comprises one of the subunits β2, β4, α2, α4 or α7 [5,8,9].

Until recently, there was significant consensus that the α5 and β3 nAChR subunits can have only an accessory or structural role in the heteropentamers in which they participate and that they are not engaged in the formation of orthosteric binding sites [10,11]. In particular, for the β3 subunit, previous studies have established that it participates in pentameric assemblies along with α4 and β2 subunits and sometimes with α6 as well, most probably forming stoichiometries such as (α4β2)_2_β3 [8,10] or (α6β2)(α4β2)β3 [10]. It has also been demonstrated that its presence influences the efficiency of assembly of nAChRs and modulates their pharmacological and electrophysiological properties. For example, when α6β4- and α6β2-containing nAChRs were co-expressed with the β3 subunit in permanently transfected HEK cell lines, an increase in calcium permeability and up-regulation by nicotine [12] was shown, indicating a significant contribution of β3 in the receptor assembly and conformational dynamics of nAChRs. Transcripts of the β3 subunit have been found mainly in the brain (i.e., cerebellum, substantia nigra, frontal cortex, hippocampus), while in other tissues they are located in testis, pancreas and pituitary glands [13] and some studies have shown the involvement of β3 in nicotine addiction [14]. β3 nAChR has not any known frequent natural variant signifying a missense mutation (highest allele frequency < 5 × 10^−3^) and none of its rare variants has been studied sufficiently [13]; thus, their physiological relevance remains elusive.

The idea that α5 and β3 are accessory subunits was recently disputed by Jon Lindstrom’s group when Jain et al. published their findings regarding the presence of unorthodox ACh binding sites in α4β2-containing nAChRs formed by α5 or β3 subunits [15], using dimeric β2α4 concatamers co-expressed with single α5 or β3 subunits. However, more recently, another group reinvestigated the possibility of an α5-contributing principal binding site using electrophysiology in pentameric concatamers of specific stoichiometry, reaching the conclusion that α5 does not participate in ACh binding, at least from its principal side [16].

Several structural studies conducted over the last two decades have uncovered various aspects of the structure–function relationships in nAChRs, such as the gating mechanism and the molecular determinants that govern the distinct pharmacology of various nAChR subtypes [2,17,18]. Starting from the low resolution structure of the *Torpedo* muscle-type nAChR [19], the advancement of the field came through several other breakthroughs such as the crystal structures of the homologous molluscan acetylcholine-binding proteins (AChBPs) [7], the high resolution monomeric ECD structures of α1 and α9 nAChRs [18,20,21], the homopentameric structure of the α2 ECD [22], the α7-AChBP chimera [23], the crystal structure of the near-intact α4β2 [24] and the most recent cryo-EM structures of α4β2 [25] and of the near-intact α7 nAChR [26,27]. Still, given the high number and complexity of the nAChR stoichiometries, in-depth understanding of the specific structural features and pharmacologic properties of the various subtypes requires further studies.

In the present study, we aimed at the elucidation of the structural features of β3-containing nAChRs in order to determine whether the structural requirements of a functional binding site are fulfilled. To this end, we solved the crystal structure of the ECD of the human β3 nAChR subunit in a monomeric state, which showed rather unique conformational features in loop C. Additionally, we pursued detailed molecular dynamics (MD) simulations in full length (α4β2)_2_β3 nAChR, using in their initial conditions our experimental β3 ECD structure, to deduce the physiological relevance of our crystallographic observations, regarding the loop C features. Finally, in another set of MD simulations we studied the stability of the protein–ligand complex when the agonist nicotine was bound to the β3 principal side (taken by its superposition with the α4 subunit of the α4β2 crystal structure [24]).

### 1.1. Crystal Structure of β3 ECD and Description of Its Unique Features

**Figure 1:**
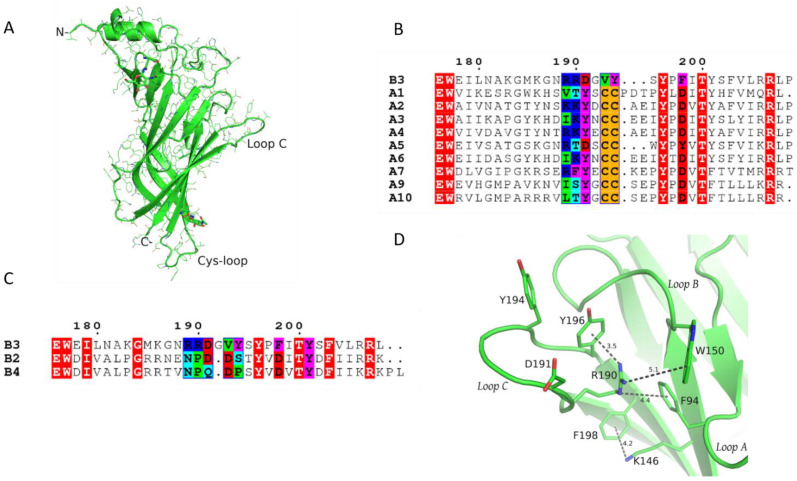
The crystal structure of β3 ECD and its sequence features. (**A**) The structure of β3 ECD retains the overall structural characteristics of all members of the pLGIC superfamily. The GlcNAC residues are shown in stick representation. (**B**) Sequence alignment of the β3 176–209 domain with the corresponding domains of all nAChR α subunits. The invariant residues are in white fonts and highlighted in red. The loop C residues of interest are highlighted in different colors, based on their physicochemical properties (positively charged residues shown in blue, negatively charged shown in red, hydrophobic shown in green, aromatic residues shown in magenta and polar residues shown in cyan). The residues lying on the tip of loop C of all α subunits are shown in orange. (**C**) Sequence alignment of the β3 176–209 domain with the corresponding domains of β2 and β4 subunits. The color coding is as in B. (**D**) The unique interaction scheme found in β3 ECD crystal structure. Arginine 190 rams the orthosteric (+) binding site being stabilized with interactions with the conserved aromatic residues.

The human β3 ECD was expressed in the yeast *Pichia pastoris* and after enzymatic deglycosylation (see Materials and Methods), the obtained crystals yielded a 2.4 Å resolution structure of its monomeric state (Table S1). As shown, the structure of β3 ECD adopts overall the same structural architecture that has been presented previously for homologous proteins [18,20,23] (Figure 1A). Its main secondary structure elements comprise the N-terminal α-helix and a ten-stranded β-sandwich core, from which several putatively functionally important loops (loops A–F) emerge. Two N-acetylglucosamine (GlcNAC) residues were clearly detected in the electron density maps, modifying predicted glycosylated asparagine residues, one at a highly conserved glycosylation site located at the C-terminal side of the Cys-loop and another at loop β5–β6 (Figure 1A). The latter is located close to the N-terminal α-helix and thus the glycan spatially occupies the same region found in other nAChR subunits, which are glycosylated at other more conserved positions instead. The loop A region adopts the same trajectory as in all other resolved Cys-loop structures despite the difference in its aromatic residue which in β3 is a phenylalanine (Phe94), as opposed to all other subunits that bear tyrosine on the corresponding position (Figure S1A, Supplementary Material). The post loop A region does not have secondary structure and in the absence of an adjacent subunit is mostly disordered, thus could only be built partially. Loop B is highly conserved and its backbone retained the common structure found in other nAChR structures.

On the contrary, loop C was found to be the most diverging region both in terms of sequence and structure with regard to the other nAChR subunits. Compared to the α subunits (Figure 1B), its length in β3 is shorter by two residues, obviously due to the lack of the vicinal cystine residues, while compared to the other two neuronal β subunits is longer by one residue (Figure 1C). The β3 subunit has the conserved positive residue (Lys186) at the pre-loop C β9-strand, and moreover, compared to the other nAChR subunits, carries excessively positively charged residues in its loop C region (Arg189 and Arg190). Although positive residues are similarly present in the corresponding positions of the α2 and α4 subunits, as was mentioned previously, the β3 loop C region is two residues shorter, intensifying further the effect of those charges (Figure 1B). Interestingly, β3 loop C lacks a functionally critical tyrosine present in all α subunits (with the exception of α5, also considered an accessory subunit), which has been replaced with aspartic acid (Asp191), while the post-loop C aspartic acid in position 198 (β3 numbering), an integral part of the gating mechanism in muscle and α9-containing nAChRs [18,28], has been replaced with a phenylalanine, adding to the overall characterization of β3 as an outlier of the nAChR family.

Unlike the β2 and β4 subunits, where at the corresponding position of 148 an arginine residue is present, the β3 subunit bears glycine, similarly to all α nAChR subunits (Figure S1B, Supplementary Material). Position 148 precedes the Ser/Thr-Trp motif of loop B and as was initially shown in the crystal structure of α4β2 nAChR, the arginine residue in β2 (β2-Arg149) sterically blocked the binding site and induced reorientation of critical aromatic residues [24]. However, as the crystal structure of β3 ECD shows, another arginine, originating this time from loop C, β3-Arg190, not present in β2 and β4, nor in α subunits, spatially places its guanidine group approximately at the same position as the guanidine group of the β2-Arg149 (Figure 1D and Figure S2A,B). This in turn induces breakage of the already deficient aromatic box, slightly reorientating the rotamer of loop A Phe94 to create a distorted sandwich cation–pi assembly with Tyr196 for the guanidine group of Arg190 (Figure 1D and Figure S2A). This unique cation–pi interaction induces further conformational shifts in the β3 loop C region. As Arg190 resides in the post β9-strand region, loop C adopts a previously unobserved trajectory with its lower and upper parts obtaining a closed-like conformation and its middle part (tip of loop C) being in an intermediate conformation (closed-in compared to β2 and more open compared to the agonist-bound α4 subunit) (Figure S2B–D).

Incorporation of the β3 ECD in the pentamer of the (α4β2)_2_β2 nAChR by performing superposition of the β3 crystal structure with any of the three β2 subunits showed that the aberrant loop C conformation in β3 ECD is completely compatible with the pentameric assembly; thus, it should not be considered indiscriminately as an artifact of its monomeric state but as having potential physiological significance. The shift of loop C due to the latter intramolecular interaction is so profound that the whole loop adopts an agonist bound-like conformation rather than a ligand-free one (Figure S2C, Supplementary Material). Therefore, the accessibility of a putative binding site formed by the (+) side of the β3 subunit for ligand molecules appears to be markedly small. Moreover, the positive charge of the Arg190 guanidine group dominates the center of the cavity, probably acting as a repellant for cholinergic ligands.

Following our previous successful co-crystallization efforts of α2 and α9 ECDs with ligands [18,21,22], we attempted in the present study either to co-crystallize β3 ECD with various ligands or to soak them in preformed β3 crystals. In either case, our efforts were proven unsuccessful since we were not able to grow ligand-bound β3 ECD crystals nor to detect clear electron density corresponding to ligands in the (+) side of the β3 ECD after the implementation of soaking experiments. Although these efforts do not exclude the possibility that β3 could bind ligands, they are indicative that under the particular experimental conditions, the β3 ECD monomer cannot contribute significantly to the binding affinity of ligands. This is in contrast to previous structural studies on α nAChR ECDs (in particular on α1 [20], α9 [18,21] and α2 [22] nAChR ECDs) where the monomeric ECD held sufficient capacity to bind strongly to common antagonists or agonists and in one case the ligand even induced the homo-pentamerization of α2 ECD [22].

### 1.2. MD Simulations of α4β2 and α4β2β3 nAChRs

**Figure 2:**
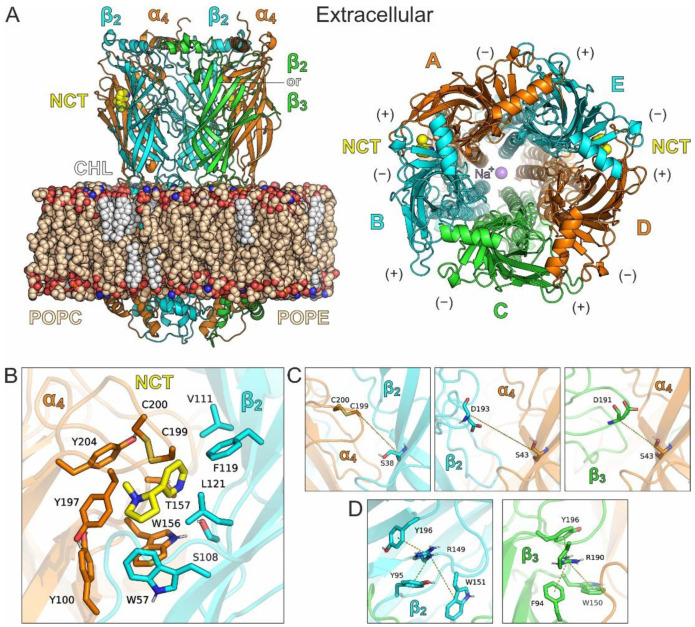
(**A**) Molecular representation of the two pentameric nAChR assemblies used for the MD simulations, excluding solvent and counter ions. The side view illustrates the initial placement of the receptor in a POPC/POPE/CHL lipid bilayer, where POPC = 1-palmitoyl-2-oleoylphosphatidylcholine, POPE = 1-palmitoyl-2-oleoylphosphatidylethanolamine and CHL = cholesterol. The top view from the extracellular side illustrates subunit arrangement, with α4 subunits as chains A and D (orange), β2 subunits as chains B and E (cyan), whereas chain C is either a β2 or a β3 subunit in the α4β2 or the (α4β2)_2_β3 receptor, respectively. The bound nicotine (NCT) at the two α4(+)/β2(–) interfaces is shown as yellow-C spheres and the purple sphere corresponds to a structural Na^+^ cation bound at the channel of the nAChRs. (**B**) Close-up view of the agonist-binding site at an α4(+)/β2(−) interface illustrating the packing of nicotine at the aromatic cage comprising Tyr100, Trp156, Tyr197, Tyr204 from α4 and Trp57, Phe119 from β2. (**C**) Definition of the loop C distance at the α4(+)/β2(−), β2(+)/α4(−) and β3(+)/α4(−) interfaces (Cα–Cα atoms). (**D**) Close-up view of the aromatic–cation interactions between Arg149 of the β2 subunit and the corresponding arrangement of Arg190 in β3.

With the aim to investigate the structural observations obtained by the X-ray structure of the ECD of β3 under physiological conditions, we have carried out classical MD simulations of pentameric α4β2-containing nAChRs comprising β3, in a lipid bilayer with explicit solvent (Figure 2A). The cryo-electron microscopy structure of the α4β2 subtype of the nAChR in 2α:3β stoichiometry (PDB ID: 6cnj) [25] was used as a template for the preparation of two systems, one with the native high sensitivity α4β2 receptor, (α4)_2_(β2)_3_, and another, where one β2 subunit was replaced by β3, producing the α4/β2/β3/α4/β2 stoichiometry (hereafter (α4β2)_2_β3).

#### 1.2.1. MD Simulations with Nicotine Bound Explicitly to α4(+)/β2(−) Interfaces

In both systems, two nicotine molecules were bound at the α4(+)/β2(−) interfaces of the ECD (Figure 2B). We performed four independent simulations of 0.5 μs and a 1 μs long simulation for each system, in order to monitor stability of the systems and adequately sample the conformational space of the receptors at this timescale (see Computational Methods). As displayed by the root-mean-square deviations (RMSDs) of the systems from the initial coordinates, the receptors displayed well-converged simulations after 100–150 ns of the MDs (Figures S3 and S4). In all simulations, the agonists bound at the α4(+)/β2(−) sites displayed high stability as a function of simulation time (Figure S5, Supplementary Material), which together with the unaltered relative positions of the transmembrane domains suggests that the two nAChRs did not alter their functional state from the crystallographically inferred desensitized state.

**Figure 3:**
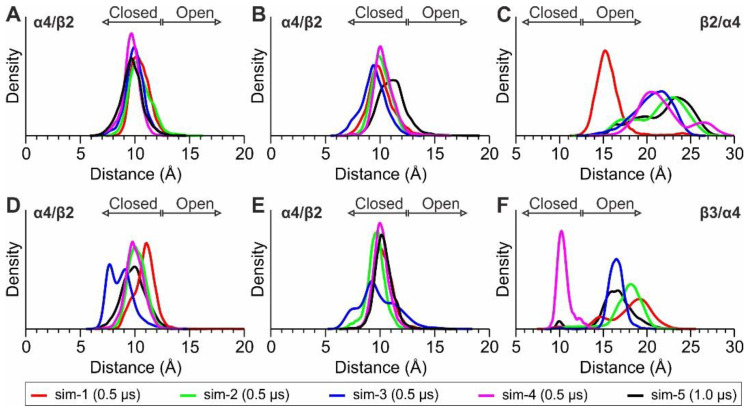
Distributions of loop C distances extracted from the MD simulations of the (α4)_2_(β2)_3_ nAChR (**A**–**C**), and the corresponding distributions from the (α4β2)_2_β3 nAChR (**D**–**F**). Panels (**A**,**B**) and (**D**,**E**) are from the two α4(+)/β2(+) interfaces of each subtype, whereas panels (**C**,**F**) are from the β2(+)/α4(−) and β3(+)/α4(−) interfaces, respectively. Loop C distances are as defined in Figure 2C and the probability density functions were calculated using the kernel estimator in R.

Considering that the opening of the loop C in α4(+)/β2(−) (distance between the tip of loop C and the conserved serine residue of the adjacent subunit, as shown in Figure 2C) has been correlated with agonist or antagonist binding [29], we calculated distributions of the corresponding distances in the two α4(+)/β2(−) interfaces and the β2(+)/α4(−) or the β3(+)/α4(−) interface of the two systems (Figure 3). In the case of the α4(+)/β2(−) interfaces, the distributions indicate a stable, closed loop C conformation in both nAChRs, a characteristic feature in agonist-bound interfaces, whereas the loop C of either β2(+)/α4(−) or β3(+)/α4(−) displayed higher variability and sampled more open states, with distances that are similar to antagonist-bound structures (>12.5 Å) [29].

**Figure 4:**
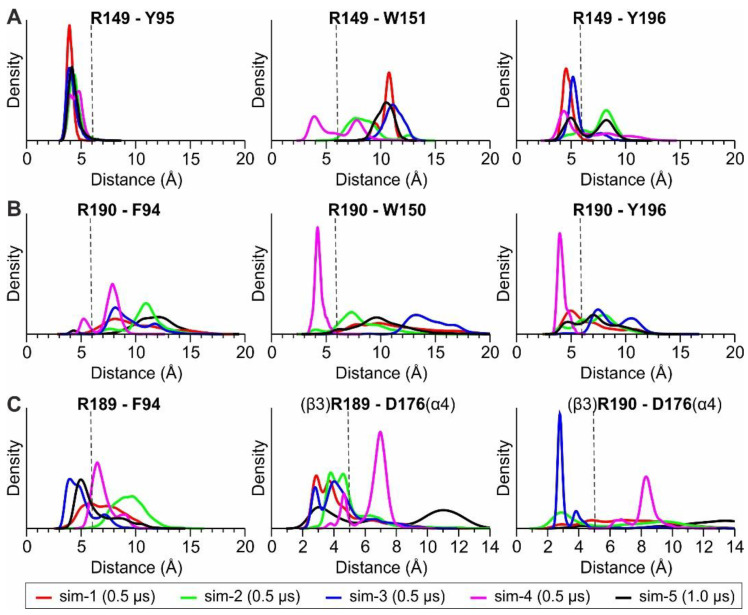
Probability density of the distances between (**A**) β2-Arg149 and the β2 conserved aromatic residues Tyr95, Trp151 and Tyr196, (**B**) between β3-Arg190 and the corresponding β3 residues Phe94, Trp150 and Tyr196, (**C**) between Arg189 or Arg190 of β3 and Phe94 of β3, or Asp176 of the adjacent α4 site. Distances were calculated between the geometric mean of the guanidinium atoms in arginine, and the aromatic atoms of the rings, or the carboxylate group of Asp176, whereas distributions were extracted using the kernel estimator in R. The dashed line indicates a proposed cut off for the distance occurring between cation–pi interacting species.

With regard to Arg190 of the β3 subunit, we monitored its distance from the three adjacent aromatic residues (Figure 2D) and compared it with the distances between Arg149 of the β2 subunit and the corresponding aromatic residues of β2. The probability distributions obtained from the MD simulations of (α4)_2_(β2)_3_ nAChR exhibit for β2-Arg149 a very stable interaction with β2-Tyr95, an interaction that is stable for most of the simulation time with β2-Tyr196 and a weak interaction with β2-Trp151 (Figure 4A). The corresponding interactions of β3-Arg190 are weaker, with only a single simulation showing stable interactions with Trp150 and Tyr196 of β3 (Figure 3B). This observation is in contrast with the stability of the interactions exhibited by Arg149 and Tyr95 or Tyr196 in β2, indicating that β3-Arg190 is more flexible than β2-Arg149. Interestingly, the adjacent β3-Arg189 displayed an interaction with β3-Phe94 for a considerable amount of simulation time (Figure 3C), an interaction that was not visible in the X-ray crystal structure. Visual investigation of the MD trajectories obtained for the (α4β2)_2_β3 nAChR revealed the high flexibility of the loop C β3 residues Arg189 and Arg190, which interchangeably place their side chain within the β3(+)/α4(−) interface (Figure S6, Supplementary Material). These simulations also revealed a potential electrostatic interaction between β3-Arg189 and α4-Asp176, which is much more probable than between β3-Arg190 and α4-Asp176 and probably indicative of a stabilizing role of β3-Arg189 for the β3(+)/α4(−) assembly (Figure 4C and Figure S6). Taken together, our MD simulations suggest that β3-Arg190 displays high flexibility within the β3(+)/α4(−) interface in contrast to the crystallographic structure of β3 ECD, where it adopted a stretched conformation, blocking the (+) binding site of β3.

#### 1.2.2. MD Simulations with an Additional Nicotine Bound to the β3(+)/α4(−) Interface

**Figure 5:**
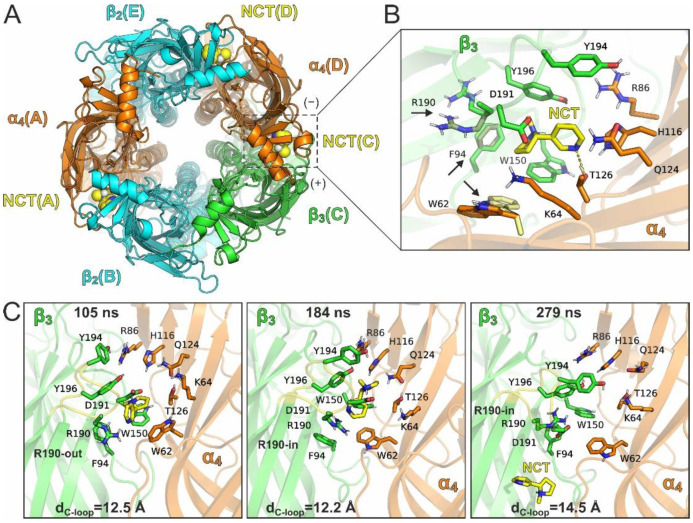
(**A**) Top view of the pentameric α4/β2/β3/α4/β2 assembly with a third nicotine molecule bound at the β3(+)/α4(–) interface (lipids and solvent are omitted for clarity). Subunits are color-coded as in Figure 2 and chain IDs are indicated in parentheses. (**B**) Close-up view of the agonist binding site illustrating the initial bound conformation of nicotine (NCT), which was taken by superposition of the nicotine-bound α4 subunit after modifying the side chain conformation of Arg190 away from the potential agonist-binding site of β3. Three starting models were employed in MD simulations by varying the initial conformation of the interacting residues that are indicated with arrows. (**C**) Snapshots from an MD trajectory illustrating nicotine dissociation from the β3(+)/α4(−) interface. These snapshots are representative structures (centroids of clusters) that were obtained by clustering of MD frames between 50 and 300 ns of the simulation (Figure S8, Supplementary Material). The close-up views illustrate only the interacting residues shown in the initial bound pose (**B**), indicating the simulation time, the state of Arg190 with respect to β3 agonist-binding site and the loop C distance (d_C-loop_) of β3(+)/α4(−).

To investigate further the potential binding of agonists at the β3 subunit, we carried out additional MD simulations of the (α4β2)_2_β3 subtype with an additional nicotine bound at the β3(+)/α4(–) site (Figure 5A). Nicotine was placed at the β3(+)/α4(−) interface in a similar orientation as between the α4(+)/β2(−) interfaces, after adjusting the side-chain orientation of β3-Arg190 away from the binding site (Figure 5B). We preferred following this methodology rather than docking the agonist to the β3(+)/α4(−) interface, because β3-Arg190 imposes non-optimal docked poses. By adjusting the side-chain of Arg190 and surrounding residues, the conformation of nicotine in our initial MD models was as favorable as within the α4(+)/β2(−) binding site. Three independent simulations were carried out starting from a different orientation of β3-Arg190, β3-Phe94 and α4-Trp62, all of which accommodated nicotine without major clashes. In all these simulations, nAChRs displayed similar stability as shown by the simulations of the receptors with two bound agonists only at the α4(+)/β2(−) sites. Both nicotine molecules at the α4(+)/β2(−) interfaces displayed high stability in all three MDs (Figure S7, Supplementary Material). However, in two of the three MDs nicotine at the β3(+)/α4(−) binding site dissociated completely after ca. 190 ns and 260 ns of simulation time (Figure S7B, Supplementary Material). In the third MD run, the β3-bound nicotine was stabilized at a conformation with RMSD > 8 Å from the initial bound pose, while the two α4-bound agonists displayed stable conformations throughout the simulation time (RMSD < 3 Å). By extracting representative structures from clustering of a trajectory that displayed agonist dissociation (Figure 5C), we observed that nicotine sampled a significant number of different orientations within the β3(+)/α4(−) interface, in most of which the loop C opening was higher (d_C-loop_ > 12 Å) than the loop C opening at the α4(+)/β2(−) binding sites (d_C-loop_ < 12 Å). In addition, β3-Arg190 displayed high flexibility with side-chain conformations either towards (R190-in) or away from (R190-out) the binding site (Figure 5C).

**Figure 6:**
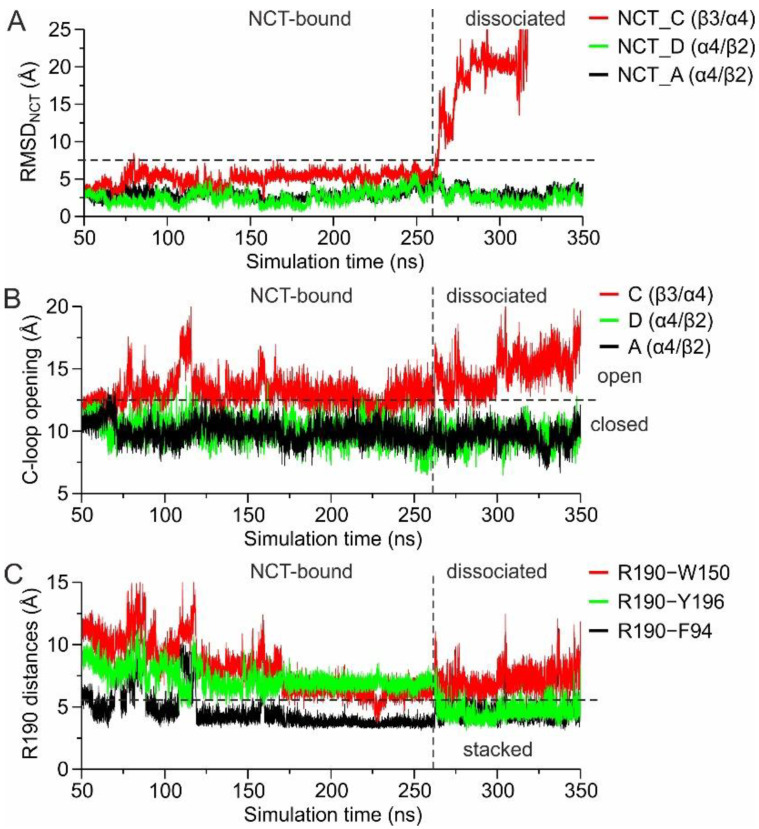
(**A**) Root-mean-square deviation (RMSD) of nicotine heavy atoms from the initial position at the three modeled interfaces of the pentameric (α4β2)_2_β3 subtype (Figure 5A), as a function of the simulation time. (**B**) The loop C distances (as defined in Figure 2C) of the three corresponding agonist-bound sites, as a function of simulation time. (**C**) Distance between Arg190 of β3(+) and the 3 interacting aromatic residues that were found in the X-ray structure, as a function of simulation time. Plots were extracted from one of the three MD simulations that exhibited nicotine dissociation as illustrated in Figure 5C (see also Figures S8 and S9).

These observations prompted us to examine the time-dependence of the events that occurred upon dissociation of the agonist in one trajectory. From the RMSD of the agonist as a function of simulation time, we observed its complete dissociation from the β3(+)/α4(−) site after 260 ns of MD simulation (Figure 6A). As long as the agonist remains bound, the opening of β3-loop C fluctuates from 12–15 Å, which upon nicotine dissociation increases further to >15 Å (Figure 6B). Strikingly and despite the high flexibility of β3-Arg190 for most of the simulation time, this residue comes into close proximity to both β3-Phe94 and β3-Tyr196 upon nicotine dissociation (Figure 6C). Taken together, these observations suggest that the flexibility displayed in the conformation of β3 loop C, accompanied by the flexibility of primarily Arg190 (and secondarily Arg189), renders β3(+)/α4(−) as an unfavorable agonist-binding interface.

## 2. Discussion

It has been more than ten years since the initial discovery of an α(+)/α(−) functional interface in the α4β2 low sensitivity subtype and since then a few more have emerged in other neuronal heteropentameric nAChR subtypes [22,30,31,32]. These findings reshaped our perception about the function of nAChRs and facilitated novel studies on selective agonists, which activate nAChRs via specific, non-classical α(+)/β(−) binding sites [33]. In this context, Jain et al. proposed the formation of functional binding sites by the β3 and α5 subunits (characterized as “unorthodox binding sites”) upon their participation in heteropentamers with α4 and β2 subunits (i.e., (α4β2)_2_β3 and (α4β2)_2_α5, respectively) [15]. In that study, the authors co-expressed dimeric α4β2 concatamers, wild type and mutated forms, with β3 or α5 subunits in *Xenopus* oocytes and conducted electrophysiology measurements which showed the presence of unorthodox ACh (+) binding sites formed by α5 or β3 subunits [15]. That was the first time that such a notion about the role of these particular nAChR subunits was published and brought controversy to the nAChR community [16,34].

Noteworthily, previous studies conducted by the same group had shown solely accessory roles of α5 and β3 subunits [10]. In these studies, HEK cells transfected with β3 along with α4–β2 concatamers upregulated nAChR surface expression by up to 25-fold and exhibited biphasic dose responses to ACh, attributed to incomplete incorporation of β3 in a population of assembled pentamers [10,35]. These types of discrepancies in electrophysiological properties of α4β2-containing nAChRs have been observed previously and have been attributed to the intrinsic properties of the different expression systems, i.e., lipidic content of cell membranes [36]. In general, using concatenated nAChR subunits has brought significant advancement in the field of nAChR function, allowing for the expression of specific stoichiometries of the naturally occurring ternary complexes in these receptors. However, recent studies have questioned whether a uniform arrangement of the concatenated nAChR subunits can be achieved by this strategy [34]. In a thorough electrophysiology study of several α4β2 concatamers expressed in *Xenopus* oocytes, Ahring et al. showed that the orientation of the assembly between concatenated subunits is highly dependent on the size of the linker connecting the successive nAChR subunits, which when being larger than six residues allows the formation of both assemblies [34]. Thus, the 18-residue-long linker used by Jain et al. [15] in the β2-α4 dimer does not necessarily induce a uniform counterclockwise assembly, but could allow for a clockwise or a mixed assembly as well (Figure S10, Supplementary Material). The introduction of this kind of ambiguity could lead to misinterpretation of otherwise robustly derived data.

While the β3 ECD has remarkable overall similarity to the other two neuronal β nAChR subunits, 77% and 78% to β2 and β4 ECDs, respectively, this is peculiarly abrogated in the region of loop C (Figure 1C and Figure S1B). More specifically, the loop C of β3 is one residue longer than the other two β subunits and is identical only in the position of Tyr196, while even their similarity is only marginally noticeable (Figure 1C). Moreover, the pre-loop B residue of β3 is glycine (Gly148), similarly to all α subunits and unlike the arginine found in β2 and β4 at the same position (Figure 1B,C). The β2 and β4 subunits are well-established participants of the (−) side in nAChR binding sites and both structural and functional studies have shown that they cannot contribute to the principal side of the binding sites [24,37]. As was shown initially by the crystal structure of α4β2, Arg149 of the β2 subunit adopts a rotamer that rams the binding site cavity, occupying a significant amount of space. As a result, its guanidine group organizes the loop C Tyr196 and loop A Tyr95 of β2 into a sandwich cation–pi assembly, displacing the loop B tryptophan residue and causing a previously unobserved spatial arrangement of the aromatic box [24,38]. In contrast, the corresponding space in all α subunits of known structure [22,24,26,27] is available for the binding of ligand molecules, a significant structural differentiation to which the absence of ACh principal sides of the binding site in β2 and β4 subunits was attributed [24,38]. The replacement of an arginine residue with glycine at the particular position of the β3 subunit (Gly148) could allow researchers to speculate that the participation of β3 as the principal component of a binding site should not be precluded.

In the present study, we determined the crystal structure of the ECD of the human β3 nAChR subunit in a monomeric state and studied its binding properties in a pentameric assembly, using thorough MDs with five independent simulations each time, covering a total simulation time of 3 μs. In particular, the crystal structure of the β3 ECD revealed unique intramolecular interactions arising from a β3-distinctive arginine residue from its loop C (Arg190), which intrudes the space of the putative binding site, organizes the aromatic residues and restricts its ability to accommodate ligand molecules (Figure 1D). Since Arg190 does not come from the core β-barrel of the β3 ECD, the conformational restrictions it imposes to the loop A phenylalanine are moderate compared to those observed in the β2 subunit (Figure S2, Supplementary Material). As a result of the interacting scheme observed herein, the whole conformation of loop C resembled the agonist-bound conformations of the α nAChR subunits more [7,22,24], despite the absence of any binding molecule. In the same line, our crystal structure indicates that the availability of space in the binding site region of loop C is rather insufficient since it is stably occupied by a positively charged loop C residue.

When these interactions were tested upon their stability in the context of the whole pentameric (α4β2)_2_β3 subtype with MD simulations, they were partially confirmed, showing engagement of β3-Arg190 with the critical loop C residue Tyr196 to a varying extent (Figure 4B). This lability was also accompanied by the adaptation of an open conformation for the β3 loop C, albeit to a lesser extent compared to the corresponding conformations found in β2 subunits (Figure 3C,F). In order to deduce whether the principal side of the β3 subunit has the capacity to bind agonists, we performed MD simulations with nicotine bound at the putative binding site of the β3(+)/α4(−) interface. Upon withdrawal of the Arg190 side-chain from this site, nicotine fitted the cavity firmly provided that its binding pose was similar to those determined in the α4β2 crystal and cryo-EM structures [24,25]. However, our MD simulations concluded that the interactions between nicotine and the β3(+)/α4(−) interface were only transient (as opposed to the nicotine bound in the α4 principal binding sites, which remained stable throughout the course of our simulations), indicating that such a binding event is rather unfavorable and therefore unlikely to occur (Figure 5). Moreover, our simulations showed that upon nicotine dissociation from the β3(+)/α4(−) interface, β3-Arg190 reoriented its guanidine group in a way to interact with the aromatic residues of β3 (Figure 5C and Figure 6C), corroborating the physiological significance of the crystallographic observations in β3 ECD monomer. These observations comply with previous studies, which had shown that the β3 subunit does not participate in functional nAChR binding sites, despite its role in modulating Ca^2+^ permeability, sensitivity to activation and nicotine addiction [8,39]. In support of that, the β3 subunit is deficient in several characteristics that the α subunits bear and seemingly are prerequisite for the formation of a principal binding site. Starting from its primary sequence, the loop C lacks a second aromatic residue (corresponding to Tyr192 in α4 subunit) which has been shown to be important in stabilizing ligands in several other nAChRs and AChBPs [7,18,21] and upon its mutation in α4 the ACh response is diminished [32,40]. Additionally, the loop A aromatic residue is phenylalanine instead of a highly conserved tyrosine (leading to a shortfall by a hydrogen bonding donor or accessor), and, maybe more importantly, the loop C is excessively positively charged, a critical drawback for any putative cholinergic binding region.

## 3. Conclusions

In the present study, we crystallized the ECD of the β3 nAChR subunit in a monomeric state and based on this structure we conducted MD simulations of the (α4β2)_2_β3 subtype, having nicotine bound at the orthodox α4(+)/β2(−) binding sites in the presence or absence of another nicotine at the β3(+)/α4(−) site. Our structural results support the belief that the β3 nAChR subunit most probably cannot participate in the formation of a binding site as its principal component, either due to stereochemical hindrance and excessive congestion of positive residues on its loop C or on account of its incompetency to accommodate agonists, such as nicotine. This is in agreement with earlier conclusions regarding the role of the β3 nAChR subunit and in contrast to more recent studies [10,15]. According to the latter ones, the β3 subunit had a functional role as a principal component of the orthosteric binding site based on electrophysiology measurements, using concatameric β2–α4 dimers mingled with a single β3 subunit. Due to the objective limitations of both structural and functional approaches leading to mis- or over-interpretations of the experimental data, we believe that further experimental structural studies, involving near-intact heteropentameric β3-containing nAChRs, are needed.

## 4. Materials and Methods

### 4.1. Construct Design

The amino acid sequence of the human nAChR β3 ECD was retrieved from UniProt (code Q05901, residues 26–232) and the expressed construct carried a single point mutation within its Cys-loop (Phe residue at position 135 was replaced with the corresponding Tyr residue present in the previously crystallized α9 ECD) in order to increase its hydrophilicity and included an N-terminal FLAG epitope and a C-terminal hexahistidine tag. The sequence was reverse-translated and codon-optimized with the Sequence Manipulation Suite [41] and the commercial program SnapGene. The synthetic DNA construct was subcloned within the first XhoI (with partial regeneration of the α-factor signal peptide) and XbaI restriction sites into the pPICZαA vector (Invitrogen) for methanol-induced expression in the yeast *P. pastoris* X33 strain (Invitrogen).

### 4.2. Protein Expression and Purification

The human β3 ECD was expressed in the yeast *Pichia pastoris*, a system already successfully used for the overexpression of several nAChR ECDs [18,21,22,42], with a yield of 8 mg/L culture. After 72 h of induction of the protein expression at 18 °C, the culture medium containing the secreted recombinant protein was collected as the supernatant of a centrifugation process (9000× *g*, 20 min, 4 °C). The medium was microfiltrated and then dialyzed against 50 mM Tris, 300 mM NaCl, 10 mM imidazole and 0.05% NaN3, pH 8.0 (buffer A) before its final concentration using the Sartorius Vivaflow 200 system. The protein was subsequently purified by affinity chromatography with Ni^2+^-NTA resin (Protino, Macherey Nagel). The β3 ECD was eluted in buffer A supplemented with imidazole to its final concentration of 200 mM and was further purified via size exclusion chromatography (SEC) on a Superdex75 increase 10/300 GL column (GE Healthcare) using as a buffer 25 mM Tris, 150 mM NaCl and 0.05% NaN_3_, pH 7.5, at a flow rate of 1 mL/min. The purified protein eluted in two or three different populations with various states of hyperglycosylation (Figures S11 and S12). The protein was enzymatically deglycosylated at 4 °C, for 72 h in the same buffer using 5 U EndoHf (NEB) per microgram of β3 ECD. Finally, the deglycosylated β3 ECD was purified again by gel filtration as described above and was further characterized with SDS-PAGE analysis (Figures S11 and S12). Interestingly, after enzymatic deglycosylation the protein eluted in a second gel-filtration step exclusively in a monomeric and monodisperse form (Figures S11 and S12).

### 4.3. Crystallization and Data Collection

Crystals of β3 ECD were grown using the sitting-drop vapor-diffusion method. As a result of random screening, crystals appeared in a condition containing 10% *w/v* PEG 6000, 100 mM Bicine pH 9.0, with a protein concentration of 2.0 mg/mL. The crystals were harvested out of their solution using LithoLoops (Molecular Dimensions) and prior to their flash vitrification in liquid N2, they were cryo-protected by immersion in a solution containing the mother liquor supplemented with 20% ethylene glycol for a few seconds. Data were collected at 100 K and at a wavelength of 0.987 Å on beamline P13 at EMBL-Hamburg, DESY, Hamburg, Germany. The reflections were integrated, merged and scaled with XDS [43], and the space group was determined with Pointless [44]. The β3 ECD was crystallized in the P21212 space group, with one molecule per asymmetric unit. Extensive efforts to acquire crystals of β3 ECD with bound small molecule ligands (i.e., ACh, nicotine, epibatidine, varenicline and cytisine) did not succeed, neither when the ligands were soaked in preformed β3 crystals nor when we attempted co-crystallization using protein to ligand molar ratios of 1:10 to 1:100. Data collection and refinement statistics are given in Table S1.

### 4.4. Structure Determination and Refinement

The β3 ECD structure was solved by molecular replacement with PHASER [45] using as a search model the structure of the human α9 nAChR ECD (PDB ID: 4UXU) [18]. The crystallographic refinement was carried out with PHENIX [46] with restrained refinement and TLS refinement implemented in the final stages. The model building and real-space refinement were performed in COOT [47] and upon completion of the refinement 99% of the residues were in Ramachandran favored or allowed regions, while 1% were outliers. The high resolution limit was determined with the CC1/2 and I/σI criteria [48] with chosen values of ~85% and 1–1.5, respectively. The program PyMOL (version 2.3, Schrödinger, LLC) was used to visualize the structures and to generate figures. The electron density of the tip of loop A could not be determined, and therefore the corresponding residues were not built in the model. The atomic coordinates and structure factors of the β3 ECD crystal structure have been deposited tothe Protein Data Bank under the accession code 8A5U.

### 4.5. Computational Methods

#### 4.5.1. Preparation of the Systems

The cryo-electron microscopy structure of the α4β2 subtype of the nAChR in the stoichiometry 2α:3β (PDB ID: 6cnj) [25] was used as template for preparation of the systems. Only chains A–E were retained and missing residues were added using MODELLER v9.24 [49]. For the α4β2β3 receptor, chain C (β2 subunit) was replaced by a β3 subunit model that was based on the X-ray structure of the ECD of β3. The structure of the full β3 subunit, including the transmembrane and intracellular domains (residues Ser1–Thr371), have been obtained using MODELLER and the coordinates of the β2 subunit (chain C) of the α4β2 receptor as template. The two bound nicotine agonists were placed at the α4 subunits (chains A and D), and a conserved sodium ion was placed at the transmembrane region. Receptors were then inserted into a lipid bilayer comprising 100:100:58 POPC:POPE:CHL lipids (where POPC = 1-palmitoyl-2-oleoylphosphatidylcholine, POPE = 1-palmitoyl-2-oleoylphosphatidylethanolamine and CHL = cholesterol) using the CHARM-GUI [50]. Systems were solvated with 33,024 TIP3P water molecules (128 waters/lipid) and the required number of Na^+^/Cl^–^ counterions to reach neutrality at ionic strength of 0.15 M. For the additional simulations of α4/β2/β3 nAChR, a third nicotine molecule was placed at the β3(+)/α4(–) interface by superimposing the nicotine-bound α4 subunit ECD (chain A) with β3 (chain C). Side-chain atoms of the β3 subunit that displayed clashes with nicotine, mainly of Arg190, were adjusted in PyMOL so as to point away from the binding site. Side-chains of the interacting aromatic residues were also adjusted in favorable orientations in the putative β3 agonist binding site. The simulation systems comprised ca. 160,000 atoms in a box with dimensions 105 × 103 × 167 (Å). Force field parameters were assigned by the LEaP module of AMBER v18 [51], using the ff14SB parameter set for the receptors [52] and the lipid14 parameter set for the lipids [53]. Parameters for nicotine were obtained using the ANTECHAMBER module with AM1BCC charges and the GAFF2 parameter set [54].

#### 4.5.2. Molecular Dynamics (MD) Simulations

All calculations were carried out using the GPU-accelerated PMEMD module of AMBER v18 [55], with the SHAKE algorithm to constrain hydrogen atoms at their equilibrium distance and an integration time step of 2 fs. Long-range electrostatic interactions were treated using the particle mesh Ewald summation method [56], with a tolerance of 10^−6^ and the real space cut-off was set to 10 Å. Temperature of the systems was regulated using the Langevin thermostat and the Berendsen weak-coupling algorithm was used to regulate pressure. Equilibration of the systems was performed in two phases after the initial energy minimization with positional restraints on the C^α^ atoms of the receptors (10 kcal∙mol^−1^∙Å^−2^) The temperature of the systems was increased to 310 K within 100 ps under constant volume (*NVT* ensemble) and 400 ps under constant pressure (*NPT* ensemble) with positional restraints of 10 kcal∙mol^−1^∙Å^–2^ on all solute atoms. Then, positional restraints on the membrane atoms were switched off and systems were equilibrated at the target temperature and pressure (*T* = 310 K, *P* = 1 atm) for 20 ns. In the second phase, positional restraints on the receptors were gradually withdrawn in 10 steps of 5 ns *NPT* simulations, after which an additional unrestrained *NPT* simulation of 30 ns was carried out (total equilibration time of 100 ns). Starting from the last frame, 4 independent *NPT* simulations were carried out for 500 ns and another one for 1 μs. The simulations of the α4/β2/β3 nAChR with 3 bound nicotine agonists have been carried out as described above, two of which were terminated after nicotine dissociation from the β3/α4 interface at 350 ns. Trajectories were processed using the CPPTRAJ module of AMBER v18 [57], and were visually investigated using VMD v1.9 [58]. Clustering of subsets of trajectory snapshots was performed using the hierarchical agglomerative approach implemented in CPPTRAJ, with a minimum distance between clusters of 1.5 Å with respect to C^α^ atoms from the ECD of the receptors. Figures were generated using PyMOL version 2.3.

## Data Availability

The atomic coordinates and structure factors of the β3 ECD crystal structure have been deposited to the Protein Data Bank under the accession code 8A5U.

## Supplementary Materials

The following supporting information can be downloaded at: www.mdpi.com/article/10.3390/molecules27144642/s1, Figure S1: presents the sequence alignment of β3 ECD with all α and β nAChR subunits; Figure S2 presents the β3(+) binding site and its comparison with the corresponding site of β2; Figure S3 presents the geometric features of pentameric nAChRs as a function of simulation time; Figure S4 presents the probability density distributions of the RMSDs of Cα atoms for the studied herein nAChRs; Figure S5 presents the probability density distributions of the RMSDs of nicotine molecules; Figure S6 presents snapshots from the MD simulations of the β3(+) site showing the flexibility of the arginine residues; Figure S7 presents plots of the RMSDs from the initial conformation as a function of simulation time; Figure S8 presents snapshots from the MD simulations with nicotine bound to the β3(+)/α4(−) binding site in several time points; Figure S9 presents snapshots from the MD simulations of the least labile nicotine bound to the β3(+)/α4(−) binding site; Figure S10 presents possible assemblies between the α4, β2 and β3 nAChR subunits; Figure S11 presents the gel filtration analysis of β3 ECD; Figure S12 presents the SDS-PAGE analysis of the purified β3 ECD and Table S1: provides the crystallographic data collection and refinement statistics.

## Sample Availability

Samples of the compounds are not available from authors.
