# Supplementary material for: Structural Insights into the Role of β3 nAChR Subunit in the Activation of Nicotinic Receptors
Source: Molecules. 2022 Jul 20;27(14):4642. doi: 10.3390/molecules27144642 (PMC9319688; doi:10.3390/molecules27144642)
Supplement: Supplementary file 1 [file molecules-27-04642-s001.zip › molecules-1799398-supplementary.pdf]

Supplementary Information

Structural insights into the role of β3 nAChR subunit in the activation of nicotinic receptors

Petros Giastas[1,2]*, Athanasios Papakyriakou[3], George Tsafaras[1], Socrates J. Tzartos[1] and Marios Zouridakis[1]*

[1]Department of Neurobiology, Hellenic Pasteur Institute, Athens, Greece

[2]Department of Biotechnology, Agricultural University of Athens, Athens, Greece

[3]Institute of Biosciences and Applications, NCSR "Demokritos", Athens, Greece

*Correspondence: Petros Giastas, petrosgiastas@aua.gr; Marios Zouridakis, marzouri@gmail.com; mzouridakis@pasteur.gr

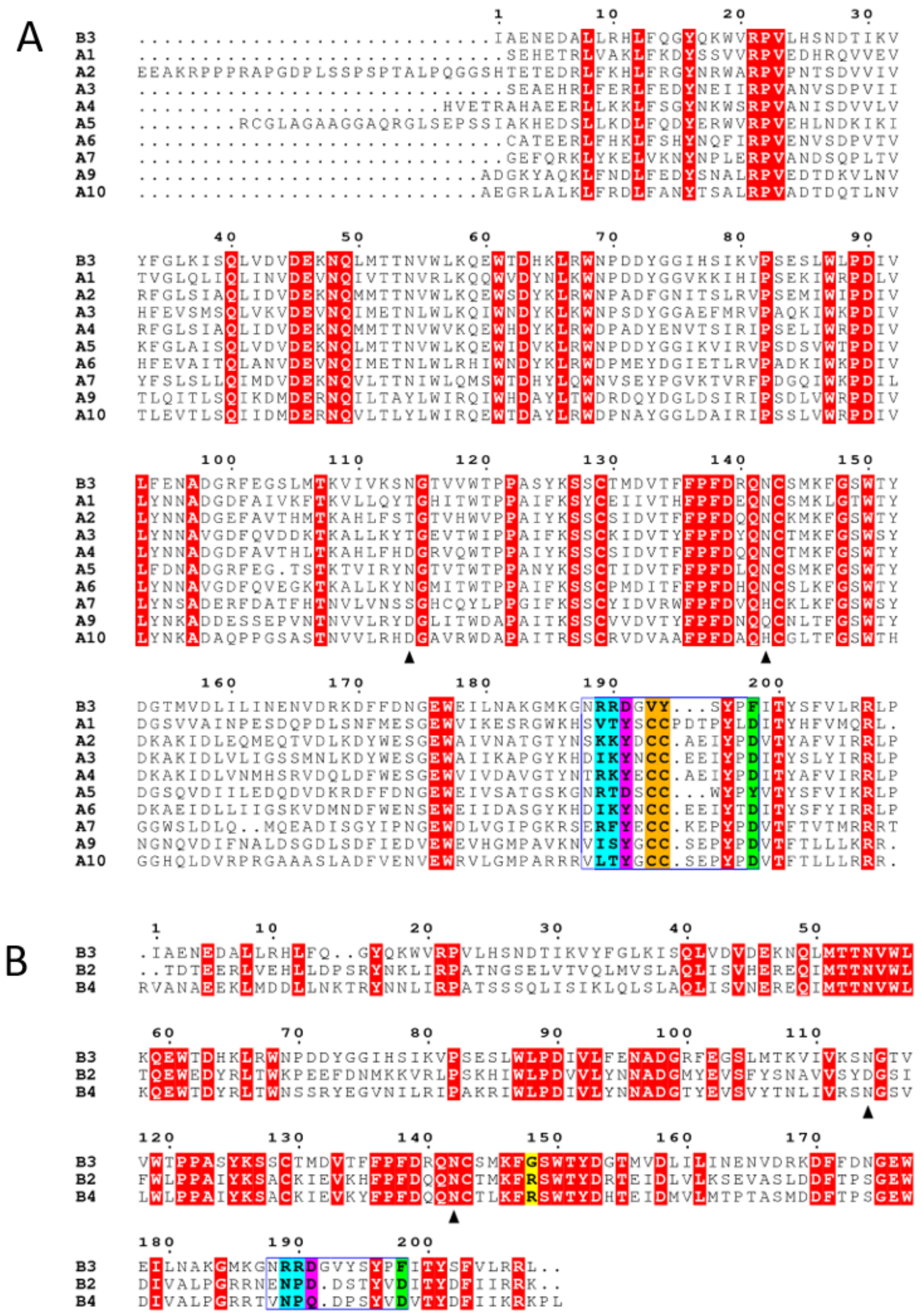

Figure S1. Sequence alignment of the β3 ECD (A) with the corresponding domains of all nAChR α subunits and (B) with the corresponding domains of β2 and β4 subunits. The invariant residues are highlighted in red. The loop C region is shown in a blue frame and the residues of interest of loop C are highlighted in different colors. The vicinal arginine residues 189 and 190 are shown in cyan, the omission of the second critical-for-function tyrosine is shown in magenta, the residues lying on the tip of loop C are shown in orange and the critical residue for the gating mechanism in α1 and α9 subunits is shown in green. The crystallized β3 ECD contains a Phe-to-Tyr mutation at position 135 (see **Construct design**). In yellow is shown the differentiation of β3 from the other two β subunits at position 148. The arrows indicate the glycosylated positions of β3 ECD.

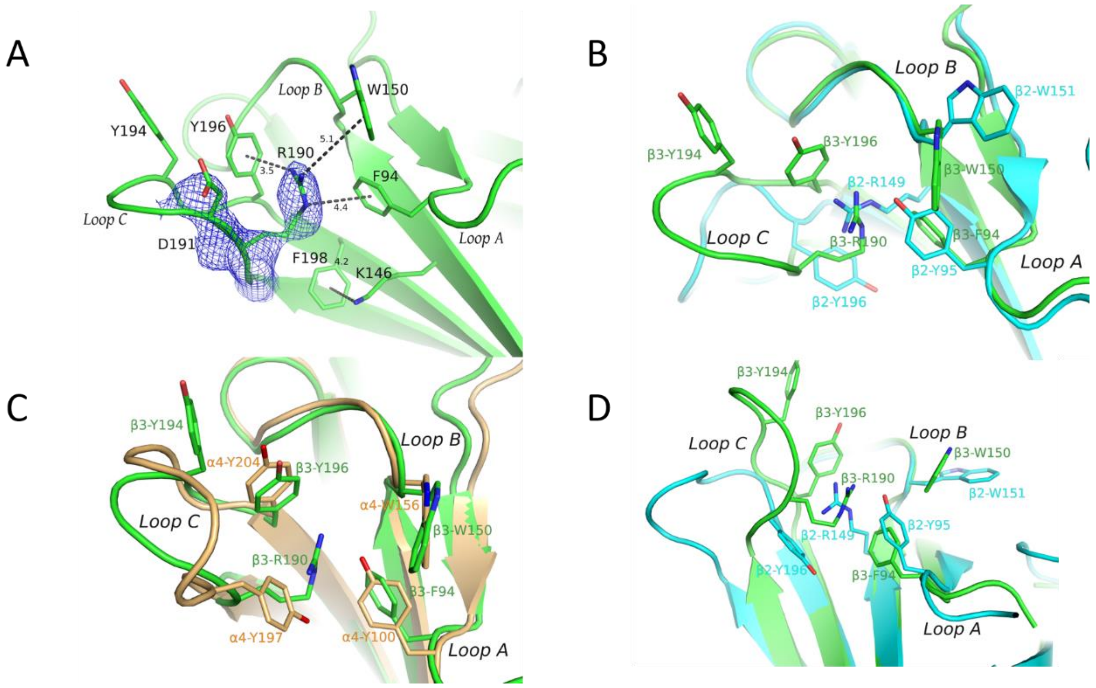

Figure S2. A) The binding site of β3 ECD displaying the electron density of Arg190 and the distances of its guanidine group from the surrounding aromatic residues. The electron density of the other residues has been omitted for clarity reasons. B) Superposition of the β3 ECD crystal structure (in green) with β2 subunit (in cyan) of the high sensitivity (HS) α4β2 cryo-EM structure[1]. The conserved aromatic residues involved in ligand binding (where applicable) and the corresponding ones of β2 and β3 ECD are shown in sticks representation. The guanidine group of β3 ECD occupies approximately the same position with the guanidine group of the β2 Arg149. The two loops C diverge substantially, likewise the other two functionally important loops, A and B. C) Superposition of the β3 ECD crystal structure (in green) with the α4 subunit (in light orange) of the HS α4β2 cryo-EM structure[1]. The intramolecular cation-π interaction of β3-Arg190 with its aromatic residues induces a closed-like conformation for most of the part of loop C. Although its diverging part includes the tip of loop C, the overall observed conformation resembles those found in agonist-bound α nAChR subunits. Loops A and B superpose well with the exception of the β3-Phe94 residue which adopts a rotamer twisted by ~70 degrees in order to interact optimally with Arg190. D) Superposition of β3 ECD with the β2 subunit shown from a lower view. The divergence of their loops C is clearly shown. The color coding is like in B.

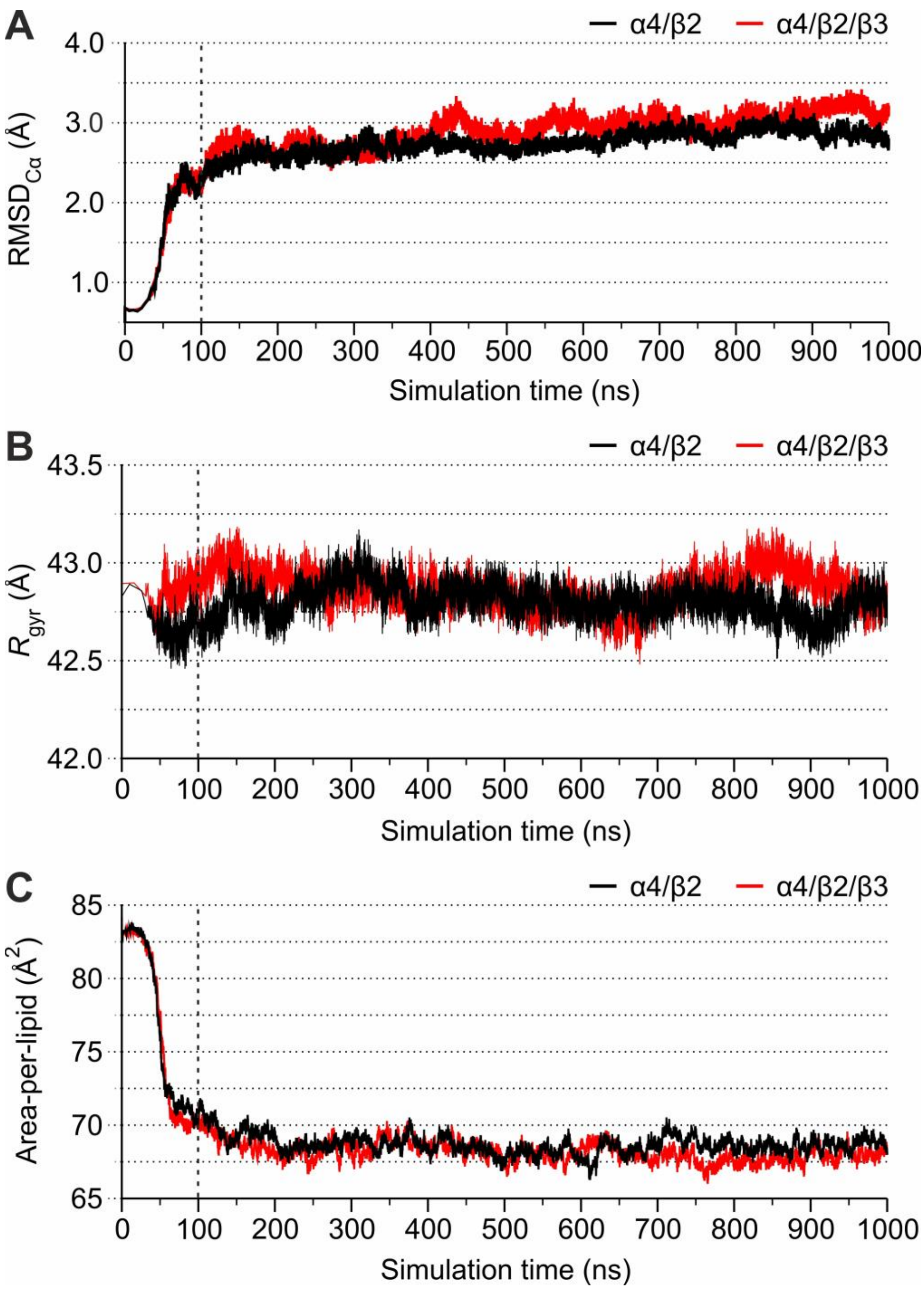

Figure S3. Plots as a function of simulation time for characteristic geometric features of the pentameric nAChRs (α4)2β2(3) (α4/β2; black lines) and (α4β2)2β3 (α4/β2/β3; red lines), displayed by the 1-µs MDs. **(A)** Root-mean-square deviation (RMSD) of all the receptor C^{α} atoms from the initial model. **(B)** Radius of gyration (R_{gyr}) of the receptor. **(C)** Mean area per lipid molecule of the system membrane (258 lipids in total). The dashed lines at 100 ns indicate the initial equilibration phase that was performed with restraints on receptor and membrane.

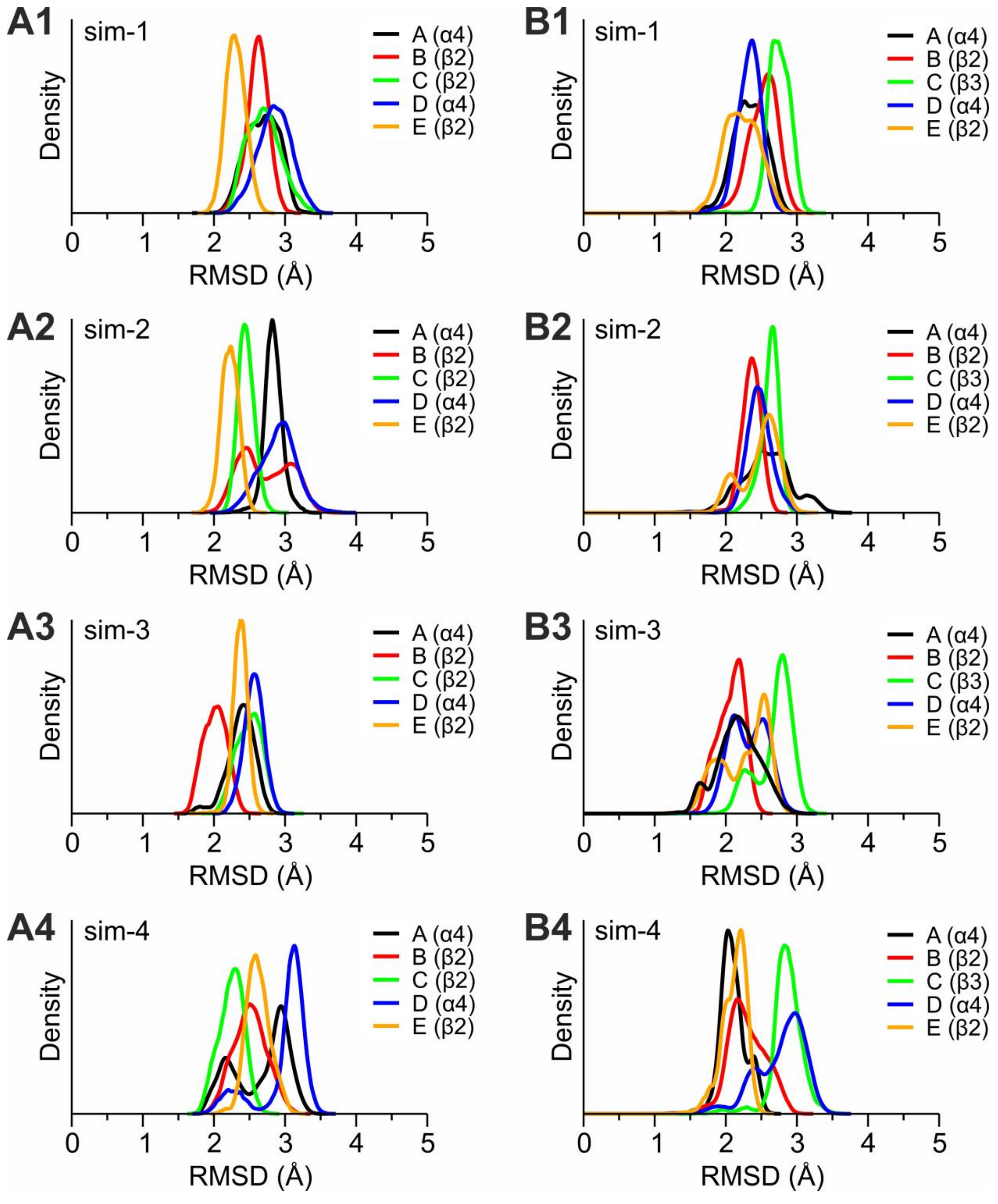

Figure S4. Probability density distributions of the root-mean-square deviation (RMSD) of C^{α} atoms of each nAChR subunit (chains A–E) from the initial coordinates, which were calculated from the 500-ns unrestraint MD simulations (sim-1–sim-4) of **(A1–A4)** (α4)2(β2)3 nAChR, and **(B1–B4)** (α4β2)2β3 nAChR.

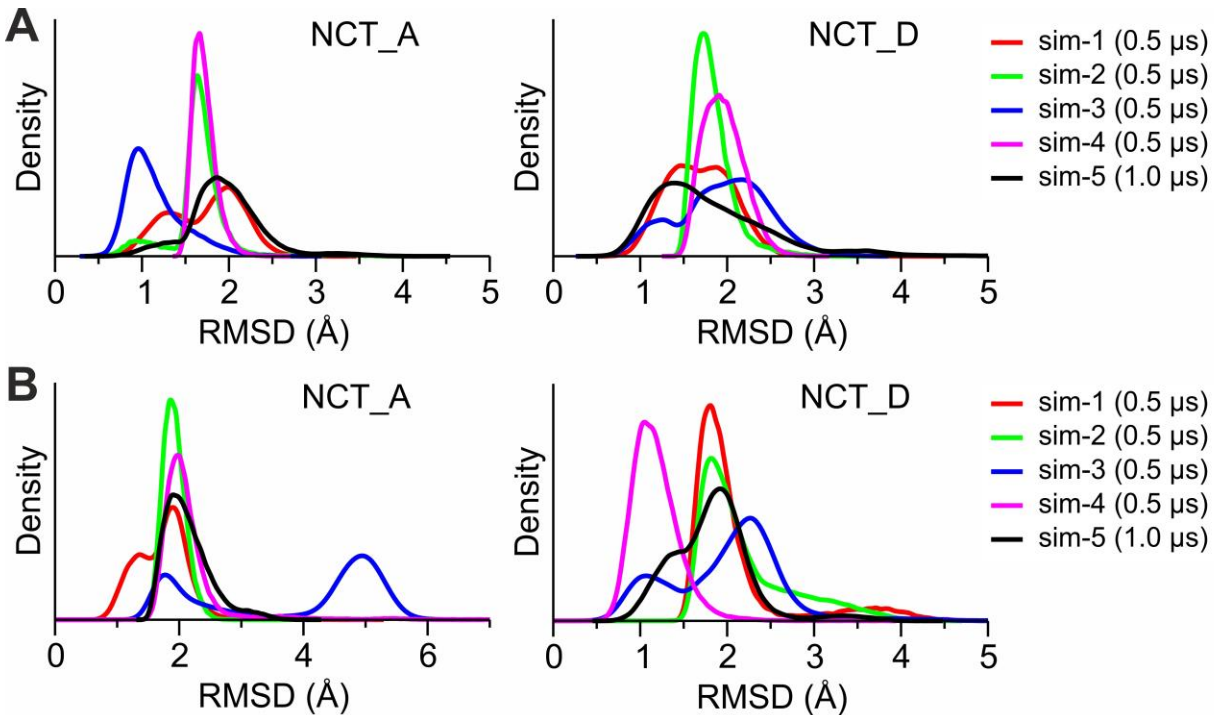

Figure S5. Probability density distributions of the root-mean-square deviation (RMSD) of nicotine heavy atoms from the initial bound pose in the two α4/β2 interfaces (chains A/B and D/E), which were calculated from the MD simulations (sim-1–sim-5) of **(A)** (α4)2(β2)3 nAChR, and **(B)** (α4β2)2β3 nAChR.

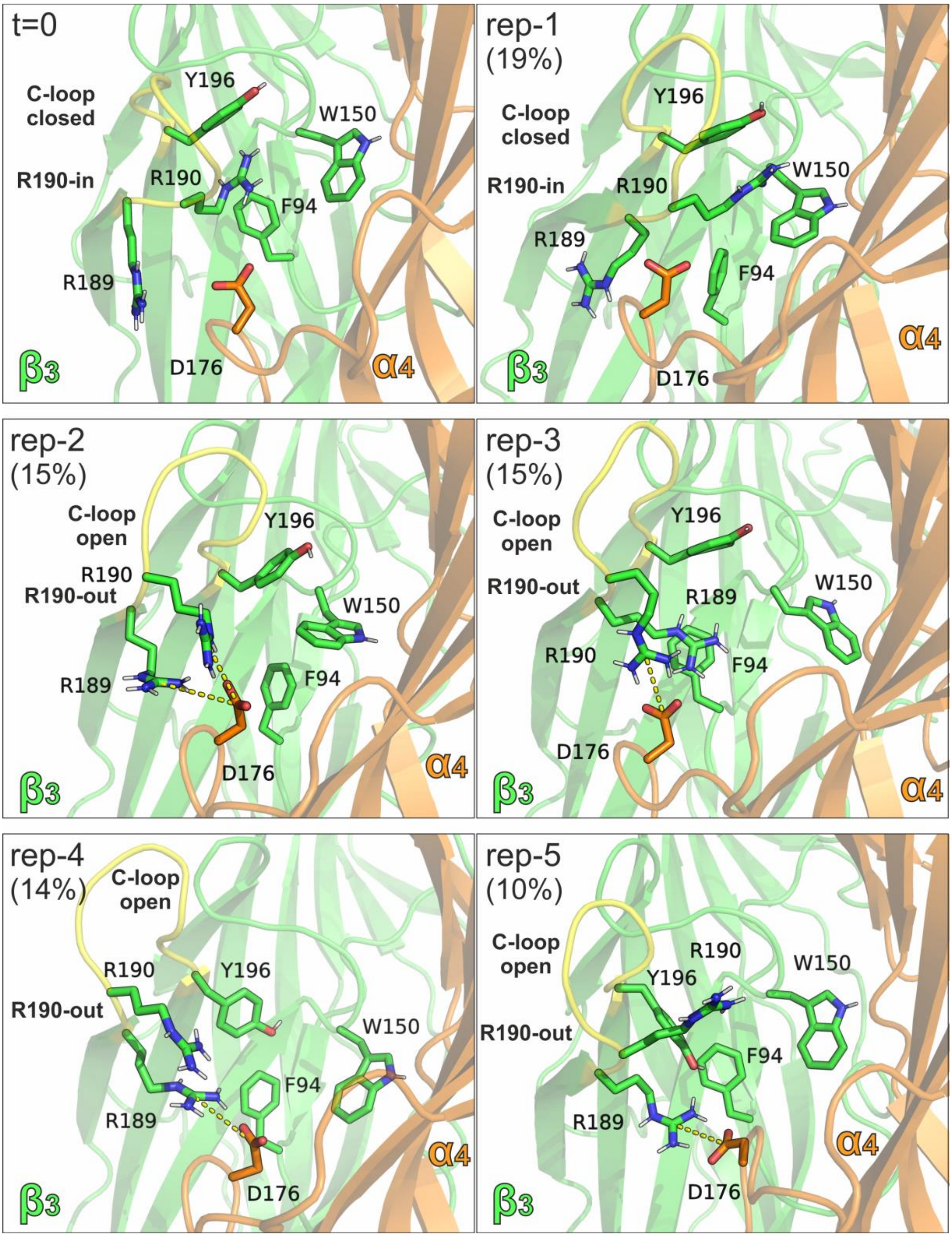

Figure S6. Snapshots from the MD simulations of (α4β2)2β3 nAChR illustrating the flexibility of the two adjacent arginine residues of β3 (Arg189, Arg190). A close-up view of the initial model (t=0) is shown along with snapshots that are taken as representative structures from clustering of MD trajectory frames (rep-1–rep-5). Values in parentheses indicate the fraction of MD frames that comprise each cluster. The C-loop is highlighted in yellow and residues are color-coded according to subunit. The yellow dashed lines indicate hydrogen-bonding, or electrostatic interactions, and the conformation of the C-loop and Arg190 is also indicated.

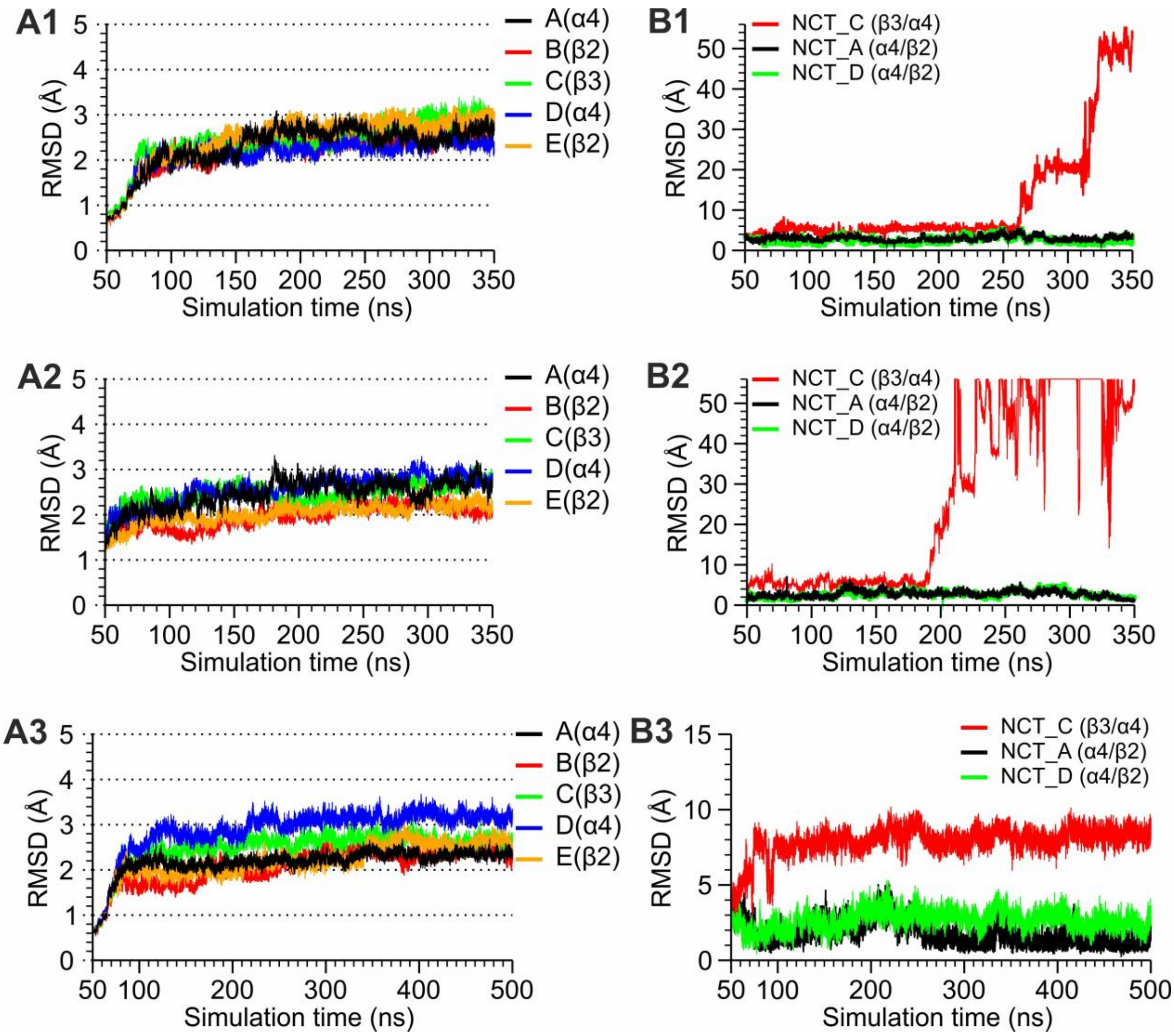

Figure S7. Plots of the RMSD from the initial conformation as a function of simulation time for (**A1–A3**) all C^{α} atoms of each subunit (chains A–E), and (**B1–B3**) all heavy atoms of nicotine molecules bound at the two α4(+)/β2(-) (NCT_A and NCT_D) and at the β3(+)/α4(-) (NCT_C) interfaces. The first two simulations were stopped at 350 ns, after complete dissociation of nicotine from β3, while the third simulation was stopped at 500 ns.

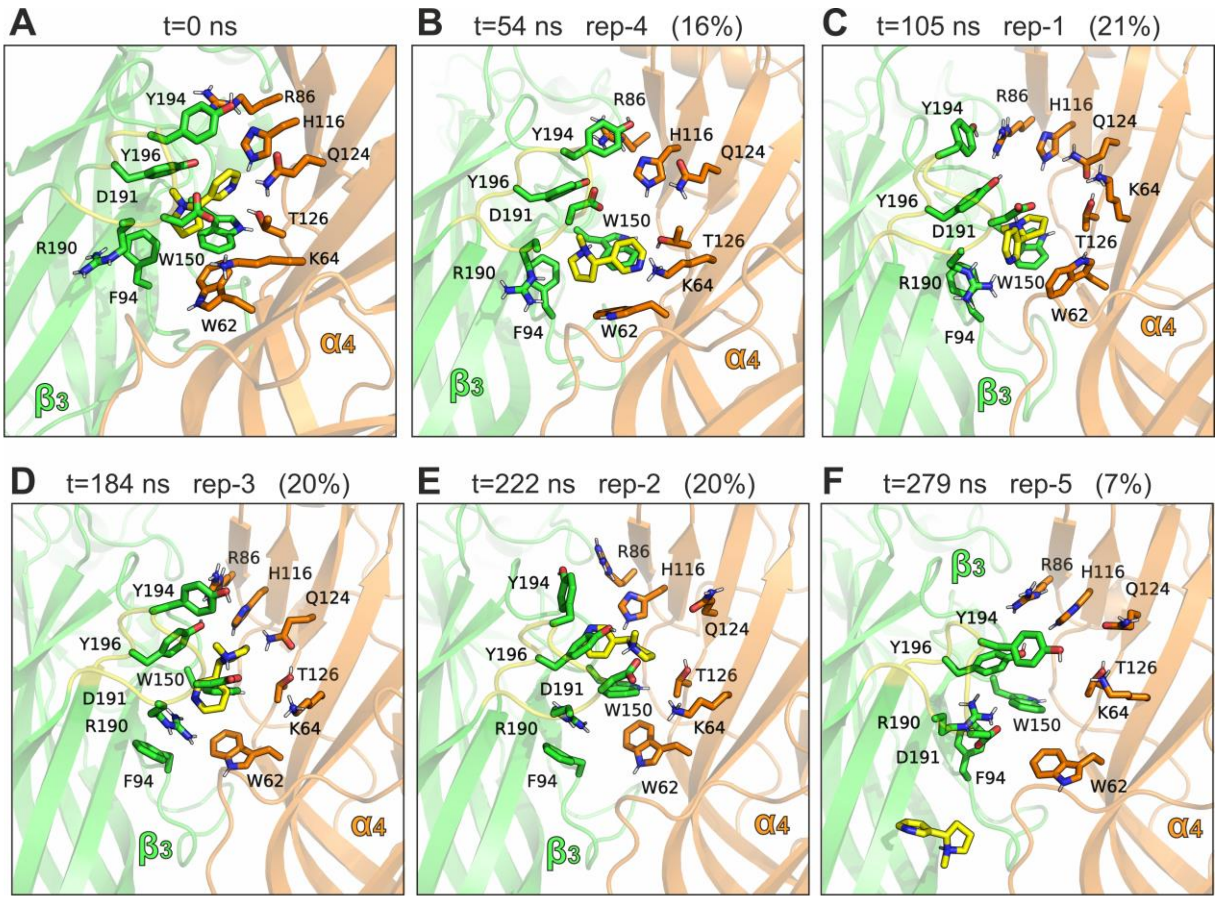

Figure S8. Snapshots taken from a 350-ns MD simulation of (α4β2)2β3 nAChR with three bound nicotine molecules. The close-up views at the β3(+)/α4(-) interface illustrate the flexibility of the bound nicotine and β3 loop C residues, and eventually, the complete dissociation of nicotine from the binding site. **(A)** Close-up view at the initial model of the β3(+)/α4(-) interface with bound nicotine. **(B–F)** Snapshots at different time points of the simulation (t in ns), which were calculated as representative structures from clustering of a fraction of the MD trajectory. The centroids (rep-1–rep-5) of the top-5 clusters are ranked according to the fraction of frames in each cluster (indicated in the parentheses).

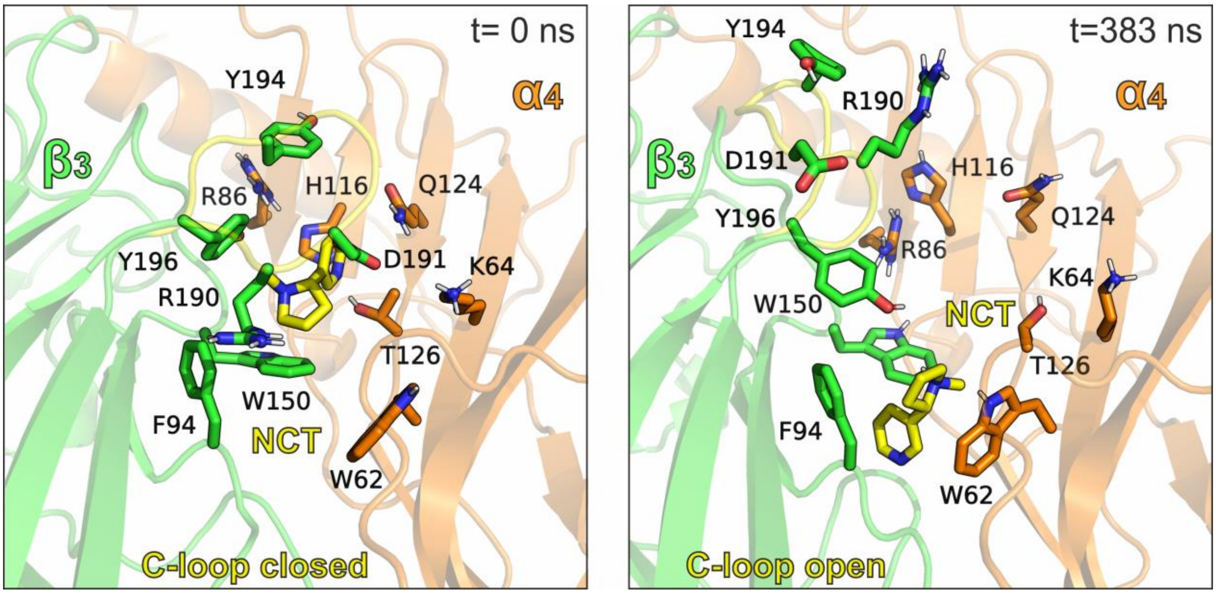

Figure S9. Snapshots taken from the 500-ns MD simulation of (α4β2)2β3 nAChR with three bound nicotine molecules. The close-up views at the β3(+)/α4(-) interface illustrate the initial bound pose of nicotine (NCT) with the C-loop of β3 closed, and a representative snapshot of the simulation where nicotine is at RMSD of ca. 8 Å from the initial pose, and is stabilized between Phe94(β3), Trp150(β3) and Trp62(α4) for a substantial time of the MD simulation. At this configuration, the loop C of β3 is mainly at an open configuration.

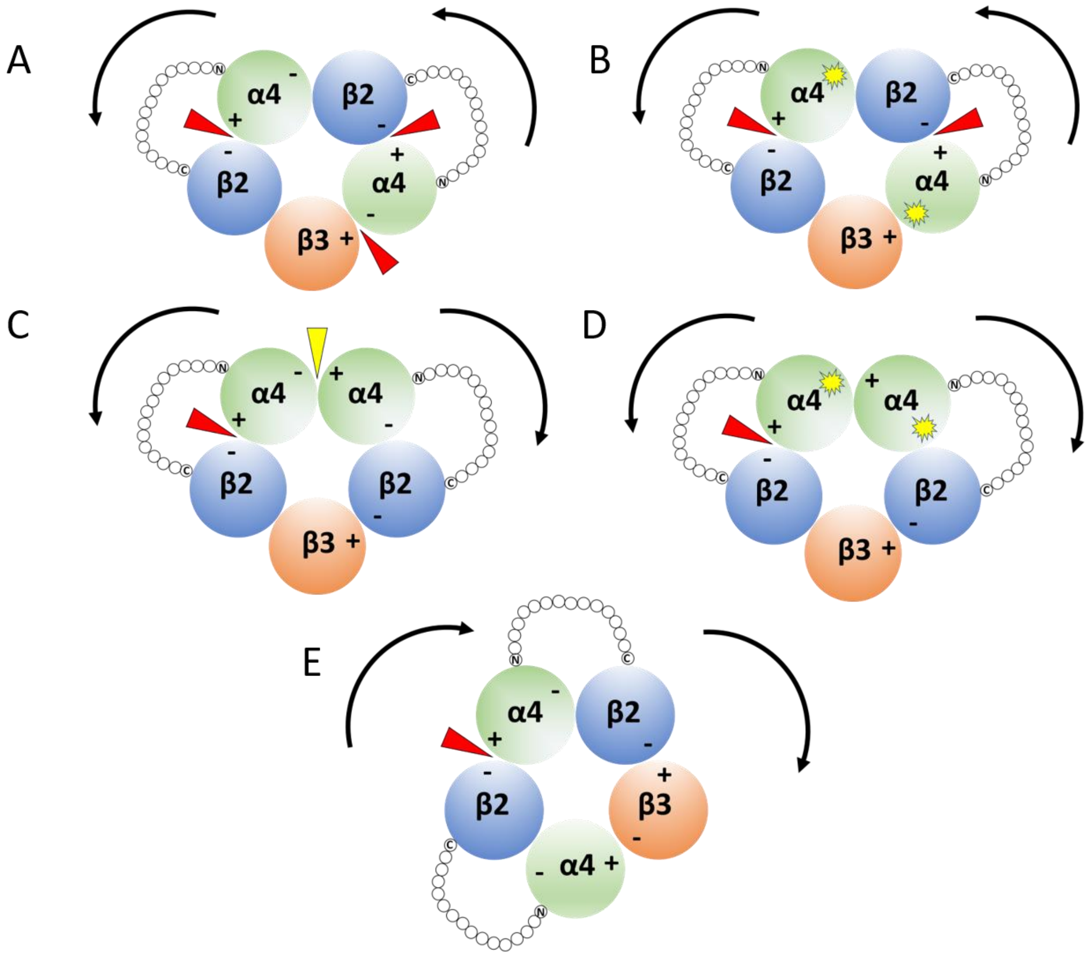

Figure S10. Schematic illustrations of possible α4, β2 and β3 subunit assemblies. A) The proposed pentameric assembly of the (β2α4)2β3 nAChR by Jain et al 2016. The particular assembly forms three equally sensitive to acetylcholine binding sites, two α4(+)/β2(-) and one β3(+)/α4(-). B) Blocking the α4(-) side with MTSET results in abolishment of the β3(+)/α4(-) binding site and thus in receptors of reduced efficacies. C) A possible mixed orientation assembly forming one α4(+)/β2(-) and one α4(+)/α4(-) binding sites. The lack of NS9283 potentiation in the electrophysiology measurements reported by Jain et al 2016 is a major discrepancy of the particular assembly. D) The mixed assembly blocked by MTSET abides to the reduced efficacy. E) A clockwise assembly leads to the formation of one α4(+)/β2(-) binding site in the presence of β3. The red and yellow triangles indicate the high and low sensitivity for acetylcholine binding sites, respectively and the yellow explosion symbol indicates the MTSET blocked α4(-) sites.

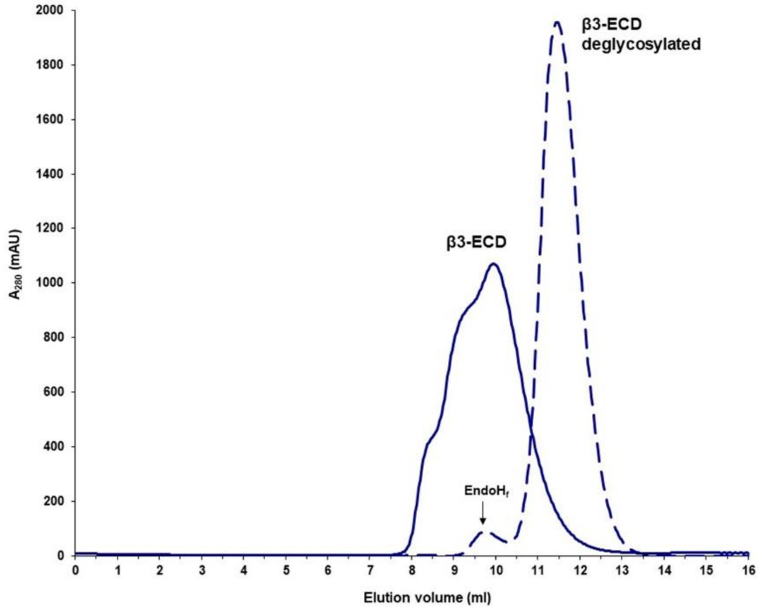

Figure S11. Gel filtration analysis of β3-ECD. Superposition of the size exclusion chromatograms of β3 ECD (solid line) and its deglycosylated form (dashed line) after treatment with EndoH$_f$, using the Superdex 75 10/300 increase column (GE Healthcare). β3 ECD was expressed with considerably high polydispersity attributed to heterogeneous glycosylation, while its deglycosylated form was eluted exclusively as a monomer with high degree of monodispersity. Fraction size was set at 0.5-ml volume and optical density (A) at 280 nm was expressed in mAU.

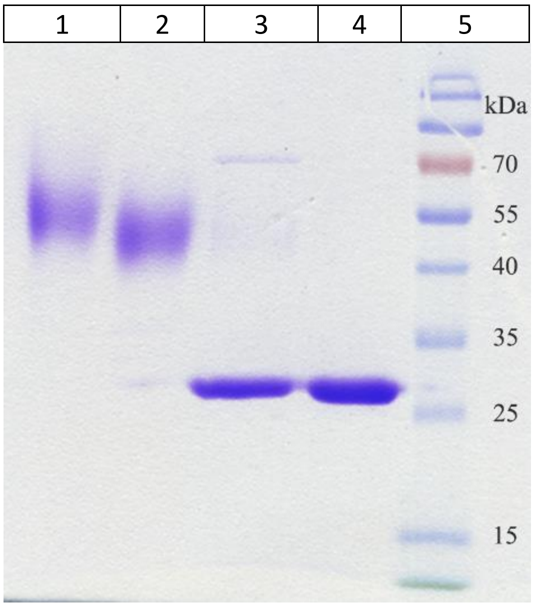

Figure S12. SDS-PAGE analysis of β3-ECD. Lanes 1-2: Glycosylated β3-ECD samples selected from the gel filtration elution volumes of 8.5 and 9.5 ml, respectively (Figure S10), revealing the heterogeneous glycosylation of the protein. Lane 3: β3-ECD after deglycosylation with 5 U/μg EndoHf for 3d at 4°C; a homogeneous population of deglycosylated β3-ECDs was retrieved at the theoretically expected molecular weight of ~27 kDa; the gel band at ~70 kDa corresponds to the enzyme used. Lane 4: Deglycosylated β3-ECD sample from the gel filtration elution volume of 11.5 ml (Figure S10). Lane 5: Protein markers of known molecular weight.

Table S1. Data collection and refinement statistics.

**Resolution range**	41.92 - 2.40 (2.49 - 2.40)
**Space group**	P 21 21 2
**Unit cell (Å, °)**	50.998, 66.772, 73.591, 90, 90, 90
**Unique reflections**	10319 (1020)
**Multiplicity**	6.1 (5.6)
**Completeness (%)**	99.86 (99.51)
**Mean I/sigma(I)**	16.33 (2.51)
**Wilson B-factor (Å^2)**	50.64
**R-merge**	0.02139 (0.2406)
**R-meas**	0.03025 (0.3403)
**R-pim**	0.02139 (0.2406)
**CC1/2**	1 (0.901)
**CC***	1 (0.973)
**Reflections used in refinement**	10307 (1020)
**Reflections used for R-free**	458 (50)
**R-work**	0.2110 (0.2823)
**R-free**	0.2816 (0.3813)
**CC(work)**	0.947 (0.861)
**CC(free)**	0.873 (0.618)
**Number of non-hydrogen atoms**	1740
**macromolecules**	1648
**ligands**	61
**solvent**	49
**Protein residues**	201
**RMS(bonds)**	0.008
**RMS(angles)**	0.97
**Ramachandran favored (%)**	96.45
**Ramachandran allowed (%)**	3.55
**Ramachandran outliers (%)**	0.00
**Rotamer outliers (%)**	7.10
**Clashscore**	18.22
**Average B-factor (Å^2)**	63.22
**macromolecules**	63.16
**ligands**	73.68
**solvent**	56.09
**Number of TLS groups**	3

Statistics for the highest-resolution shell are shown in parentheses.

References

1. Walsh Jr., R. M. *et al.* Structural principles of distinct assemblies of the human alpha4beta2 nicotinic receptor. *Nature* **557**, 261–265 (2018).
